# High Molecular Weight Polymer Promotes Bone Health and Prevents Bone Loss Under *Salmonella* Challenge in Broiler Chickens

**DOI:** 10.3389/fphys.2018.00384

**Published:** 2018-04-13

**Authors:** Sandi Raehtz, Billy M. Hargis, Vivek A. Kuttappan, Rifat Pamukcu, Lisa R. Bielke, Laura R. McCabe

**Affiliations:** ^1^Department of Physiology, Michigan State University, East Lansing, MI, United States; ^2^Department of Poultry Science, University of Arkansas, Fayetteville, AR, United States; ^3^Midway Pharmaceuticals, Spring House, PA, United States; ^4^Department of Animal Science, Ohio State University, Columbus, OH, United States; ^5^Department of Radiology, Michigan State University, East Lansing, MI, United States; ^6^Biomedical Imaging Research Centre, Michigan State University, East Lansing, MI, United States

**Keywords:** bone, osteoporosis, poultry, microbiome, intestine, gut, *Salmonella*, femoral-neck

## Abstract

As a consequence of rapid growth, broiler chickens are more susceptible to infection as well as bone fractures that result in birds being culled. Intestinal infection/inflammation has been demonstrated to promote bone loss in mice and humans. Given this link, we hypothesize that therapeutics that target the gut can benefit bone health. To test this, we infected broiler chickens (7 days old) with *Salmonella* and treated the birds with or without MDY, a non-absorbable mucus supplement known to benefit intestinal health, from day 1–21 or from day 14–21. Chicken femoral trabecular and cortical bone parameters were analyzed by microcomputed tomography at 21 days. Birds infected with *Salmonella* displayed significant trabecular bone loss and bone microarchitecture abnormalities that were specific to the femoral neck region, a common site of fracture in chickens. Histological analyses of the chicken bone indicated an increase in osteoclast surface/bone surface in this area indicating that infection-induced bone resorption likely causes the bone loss. Of great interest, treatment with MDY effectively prevented broiler chicken bone loss and architectural changes when given chronically throughout the experiment or for only a week after infection. The latter suggests that MDY may not only prevent bone loss but reverse bone loss. MDY also increased cortical bone mineral density in *Salmonella*-treated chickens. Taken together, our studies demonstrate that *Salmonella-*induced bone loss in broiler chickens is prevented by oral MDY.

## Introduction

In the US, in 2010, approximately 36.9 billion pounds of broilers were sold for a retail value of 45 billion dollars and 6.8 billion pounds were exported (according to uspoultry.org). Over the years, chickens have been bred to grow rapidly to quickly obtain large muscle mass to increase market weight. This rapid growth has contributed to bone abnormalities, deformities and fractures (Vestergaard and Sanotra, 1999; Butterworth et al., 2001; Kestin et al., 2001; Knowles et al., 2008; Olkowski et al., 2011). The most common site of bone pathology is at the femur, specifically the femoral neck, which results in bird lameness (McNamee et al., 1999; McNamee and Smyth, 2000; Dinev, 2009; Durairaj et al., 2009; Olkowski et al., 2011). Selection for high growth rates is also linked to the development of chickens with decreased immune function which puts the birds at increased risk for bacterial infections (McNamee et al., 1999; Corr et al., 2003). Bacterial infections and bone loss/degeneration are major health concerns in broiler chickens that lead to economic losses of hundreds of millions of dollars annually (Cook, 2000).

Intestinal infection and it's associated inflammation are known to promote bone loss in mouse models and in patients with inflammatory bowel disease (Lin et al., 1996; Sylvester, 2005; Hamdani et al., 2008; Harris et al., 2009; Irwin et al., 2013, 2016; Sylvester et al., 2013). Studies in mice indicate that the extent of intestinal inflammation directly correlates with bone density loss (Irwin et al., 2016) and is linked to suppressed bone formation (Harris et al., 2009; Irwin et al., 2013). Correspondingly, removing the mediator of intestinal inflammation results in bone density recovery (Harris et al., 2009). In avian models, the link between enteric inflammation and bone health has not been directly examined, but several reports indicate an association (Huff et al., 2006; Tellez et al., 2014; Wideman, 2016; Bielke et al., 2017). For example, broiler chickens fed a rye diet display mucosal damage, increased intestinal viscosity and permeability, and lowered bone strength and mineralization (Tellez et al., 2014). Rapidly growing turkeys exhibit altered stress responses and increased chronic bacterial diseases such as turkey osteomyelitis complex (Huff et al., 2006) similar to rapidly growing broiler chickens which display altered immune function, bacterial infection and bone deformation (McNamee et al., 1999; Corr et al., 2003). Pro-inflammatory response are associated with bone loss in many species including broiler chickens (Mireles et al., 2005). Thus, broiler chickens are at increased risk for infection and possibly decreased bone health as a consequence of pressures for increased growth, large-production facilities, and decreased antibiotic use (Cook, 2000; Mehaisen et al., 2017).

*Salmonella* infection in chickens can cause mild enteric inflammation characterized by increased mRNA expression of proinflammatory cytokines including interleukin-6 (IL-6), IL-8, IL-12, LPS-induced tumor necrosis alpha factor (LITAF), and interferon gamma (IFN-γ) (Kaiser et al., 2000; Withanage et al., 2005; Haghighi et al., 2008; Quinteiro-Filho et al., 2010, 2012; Higgins et al., 2011; Setta et al., 2012; Kubota et al., 2013). Salmonella infection in mammals also causes inflammation (Eckmann and Kagnoff, 2001), however the specific cytokine responses are quite different between rodents and chickens. Ingestion of selected probiotics, which can promote immune quiescence in the intestine, can reduce intestinal inflammation (Haghighi et al., 2008; Neish, 2009). In fact, a commercially used poultry probiotic selected for anti-*Salmonella* activity has been shown to induce changes in expression of genes related to lipopolysaccharide (LPS) response, NF-κB, and apoptosis pathways *in vivo* and *in vitro* (Higgins et al., 2011; Carey and Kostrzynska, 2013) in response to *Salmonella typhimurium* challenge. The exact factors that cause this effect are not yet known.

MDY (MDY-1001, Midway Pharmaceuticals) is a non-absorbed, non-metabolized derivative of a high molecular weight polyethylene glycol (PEG) polymer averaging 15 kDa in size. PEG derivatives are hydrophilic molecules which have been used in medicine for several years. Short chain length PEG molecules have oncotic properties that are favorable for absorbing water molecules and reducing tissue edema as well as acting as a reservoir for radical oxygen species (ROS) preventing cell membrane damage (Koob and Borgens, 2006; Luo and Shi, 2007). Because of these properties, PEG derivatives have been used in bowel prep solutions (GoLytely; Braintreelabs) and pharmaceutical drug delivery (pegylated compounds). Additionally, certain PEG molecules can increase epithelial cell tight junctions and mucus barrier function resulting in a preserved epithelial cell barrier (Moeser et al., 2008; Teramura et al., 2008; Valuckaite et al., 2009; Oltean et al., 2012, 2015). High molecular weight PEGs, like MDY, can associate with the lipid rafts of cell membranes (Valuckaite et al., 2009). They can also prevent pathogenic bacteria (*Pseudomonas aeruginosa)* adherence to the epithelial barrier and prevent subsequent sepsis and death in mice (Wu et al., 2004). High molecular weight PEG has also been shown to promote organ transplant preservation and prevent radiation induced mucosal damage (Valuckaite et al., 2009, 2013; Oltean et al., 2012, 2015).

*Salmonella* is known to infect mouse and human osteoblasts, cause bone and joint infection (osteomyelitis) clinically (Alexander et al., 2001; Marriott, 2013), promote inflammatory bone loss (Marriott, 2013), and promote enteric inflammation (Kaiser et al., 2000; Eckmann and Kagnoff, 2001; Withanage et al., 2005; Haghighi et al., 2008; Higgins et al., 2011; Quinteiro-Filho et al., 2012; Kubota et al., 2013). While the above studies report effects of paratyphoid *Salmonella*, several studies have reported effects of a specific *Salmonella* serovars, *Salmonella Enteritidis*, in chickens (Kogut et al., 1994, 1995; Stabler et al., 1994; Kaiser et al., 2000). Based on the link between gut and bone health (Mccabe et al., 2013; McCabe et al., 2015; Irwin et al., 2016; Collins et al., 2017a,b; Rios-Arce et al., 2017; Schepper et al., 2017), we hypothesized that *Salmonella* infection could have negative effects on broiler chicken skeletal health. Furthermore, given the effect of high molecular weight PEGs in strengthening the intestinal barrier (Teramura et al., 2008; Valuckaite et al., 2009, 2013; Oltean et al., 2012, 2015), we hypothesized that MDY may benefit the bone health of broiler chicks infected with *Salmonella*. Our studies support this premise.

## Methods

### Experimental design

**Figure 1:**
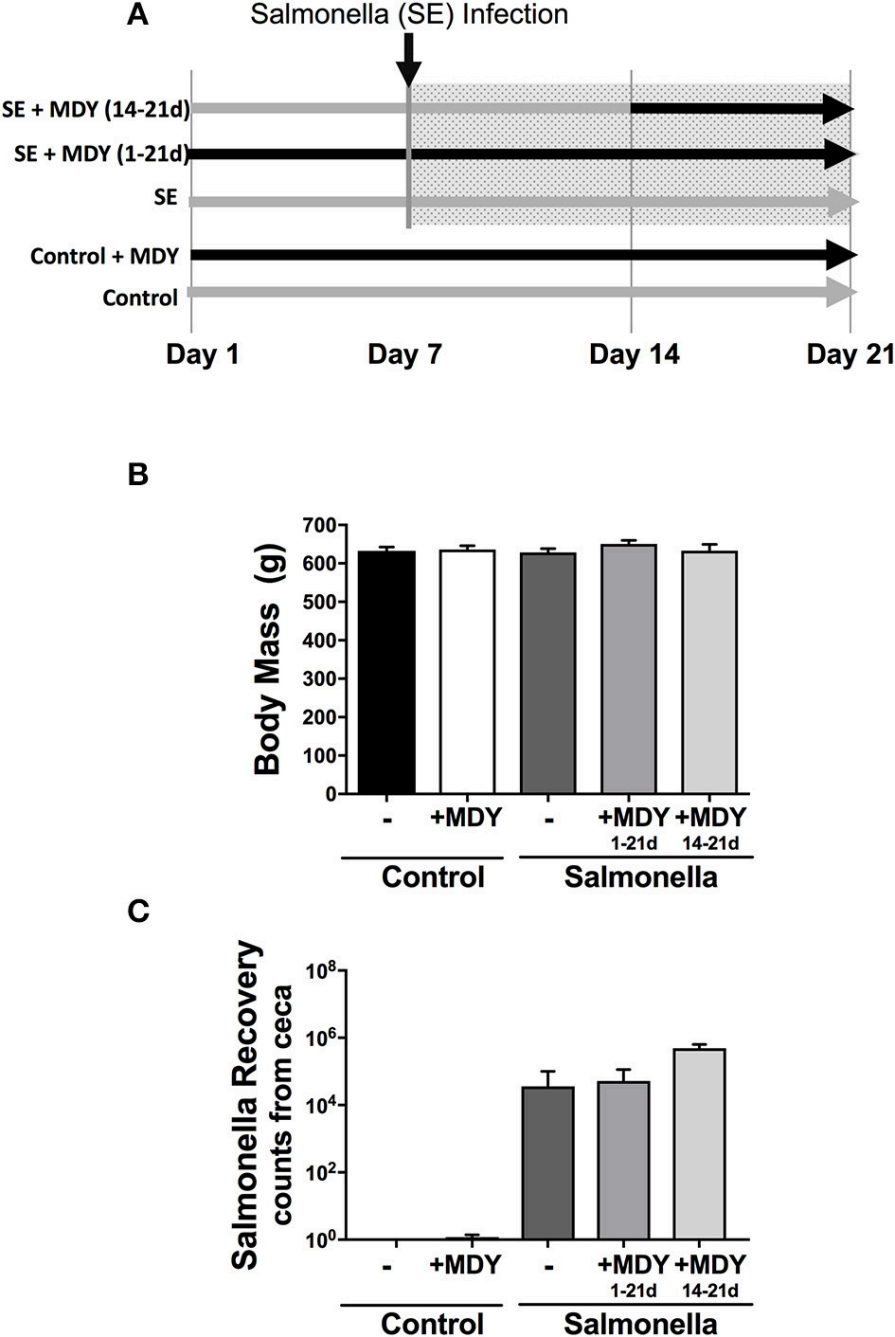
Experimental design and chicken body mass. **(A)** 1-day-old chicks were weighed and evenly distributed into 5 groups: Control, control + 0.33% MDY in feed (1-21d), *Salmonella*-infected, *Salmonella*-infected + 0.33% MDY in feed (1-21d), and *Salmonella*-infected + 0.33% MDY in feed (14-21d). The birds were harvested on day 21. **(B)** Chick body mass was measured on experimental day 21. **(C)** Salmonella recovery rates from chicken ceca. Values represent averages ± SE. *N* ≥ 8.

Broiler chickens at 1-day-old were equivalently weight-distributed into the following treatment groups: (1) control (*n* = 24), (2) control + MDY for 3-weeks (*n* = 24), (3) *Salmonella*-infected (*n* = 24), (4) *Salmonella*-infected + MDY for 3-weeks (*n* = 24), and (5) *Salmonella*-infected + MDY for 1-week (*n* = 15). For all *Salmonella* groups, birds at day 7 were infected by gavage with *Salmonella Enteritidis* (a total of 10^6^ colony forming units (cfu) in 0.25 ml). For MDY treatment groups, birds were given *ad libitum* feed containing 0.33% MDY starting either on day 1 (3-week treatment; days 1 through 21; groups 2 and 4) or on day 14 (1-week treatment; only days 14-21; group 5) (Figure 1A). MDY (MDY-1001) is a high molecular weight PEG derivative being developed and generously provided by Midway Pharmaceuticals. Specifically, MDY is non-immunogenic and composed of 2 PEG chains crosslinked by a phenolic linker. The average MW of the MDY is 15 kDa. On day 21 of the study, the birds were humanely killed by carbon dioxide inhalation. Legs were removed at the hip joint and the femora isolated and fixed in formalin for 1 week and then transferred to 70% ethanol. All animal handling procedures were in compliance with the Institutional Animal Care and Use Committee at the University of Arkansas.

### Salmonella culture

A primary poultry isolate of *Salmonella Enteritidis* (SE), phage type 13A, was used to infect the chickens. This strain is associated with decreased performance and increased enteric inflammation (Higgins et al., 2011; Quinteiro-Filho et al., 2012). The bacterium was originally obtained from the National Veterinary Services Laboratory (Ames, Iowa). For the study, *Salmonella* was grown in tryptic soy broth for approximately 8 hours. The cells were washed three times with 0.9% sterile saline by centrifugation (1,820 × g), and the approximate concentration of the stock solution was determined spectrophotometrically. The stock solution was serially diluted and confirmed by colony counts of three replicate samples (0.1 mL/replicate) that were spread plated on tryptic soy agar plates.

### Microcomputed tomography

**Figure 2:**
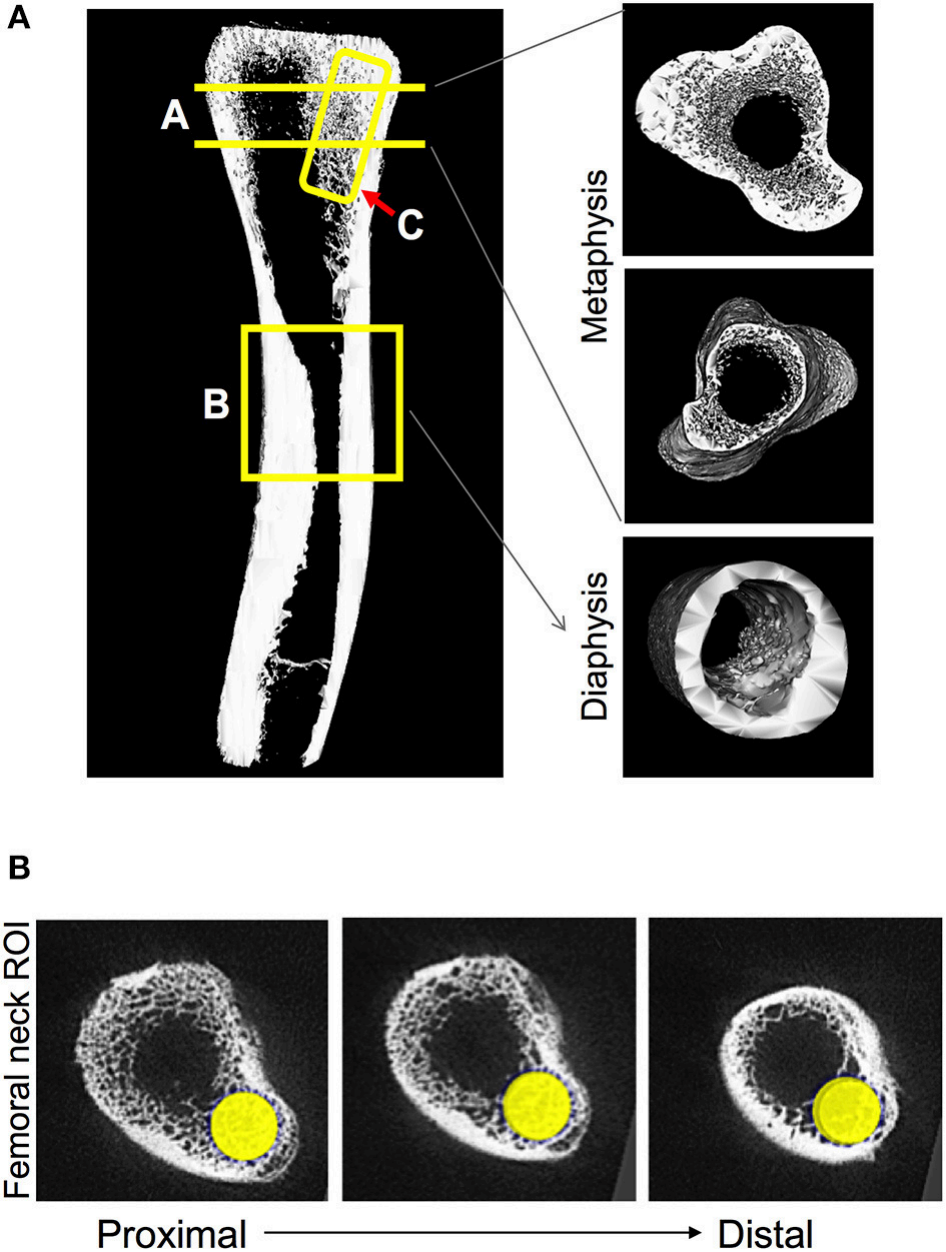
Regions of chicken femoral bone analyzed by microcomputed tomography. **(A)** Isosurface image indicating regions of bone examined: A-Region = trabecular bone in the metaphysis that is measured at 10% of the total femur length starting from the growth plate and moving distally. B-Region = diaphyseal cortical bone that is measured at 50% of the total length of the femur by a 7 mm cube region of interest. C-Region = femur neck trabecular bone region of interest (ROI) measured by utilizing a 2 mm diameter × 4 mm length cylindrical tube. **(B)** Three sections, proximal-to-distal, indicating the location of the cylinder used for the femur neck ROI.

Femora were scanned using a GE Explore Locus microcomputed tomography system with a voxel resolution of 20 μm obtained from 400 views. Beam strength was set at 80 kV and 450 μA with a beam angle increment of 0.5. Each scan consisted of 2 femurs placed in a 50 mL tube filled with ethanol and were scanned with a phantom bone used for calibration between scans. A fixed threshold of 376 was used to separate bone from bone marrow. Regions of interest (ROI) were identified and analyzed for bone density parameters and isosurface images were constructed using GE Healthcare Microview software. Three areas of the femur were focused on: proximal femoral metaphysis, femoral diaphysis, and femoral neck as noted in Figure 2. The length of the bone was calculated and trabecular bone was measured for 10% of the total length of the bone starting at the growth plate and moving proximally. Femurs were reoriented so that the femoral neck was along the y axis and a cylindrical tube (2 mm diameter × 4 mm length) was used to analyze trabecular bone starting at the growth plate and going 4 mm down toward the proximal end of the bone. Finally, cortical bone shape/density was measured by placing a 7 × 7 × 7 mm cube at the diaphysis at 50% of the total bone length. In addition, a 0.75 mm cube was used to measure only cortical bone in this region.

### Histology

Femurs, fixed in formalin and subsequently transferred to 70% ethanol, were decalcified by soaking in 14% EDTA for 9 days followed by a 2 h rinse with running tap water. Femurs were then processed on an automated Electron Excelsior tissue processor (Thermo Fischer Scientific) for dehydration, clearing and infiltration using an extended schedule secondary to size. Samples were embedded by hand on a flat surface to demonstrate a longitudinal section through the femoral neck and proximal femur. Midway down, the bone shaft was sectioned transversely for cross section of bone as well as bone marrow analysis. Paraffin blocks were sectioned at 4 μm on a Reichert Jung 2030 rotary microtome (Leica, Wetzlar, Germany).

Osteoclast, osteoblast and adipocyte quantification was performed by staining for tartrate-resistant acid phosphatase (TRAP) activity with a hematoxylin counterstain per manufacturer protocol (387A-IKT; Sigma). At least 10 images at 40X magnification were taken at the femoral neck, excluding the growth plate area, for osteoclast and osteoblast quantification and at least 5 images at 20 X magnification were taken of the bone marrow for adipocyte measurements. Total bone surface measured averaged 1 mm or greater per image and did not differ between groups (data not shown). TRAP-positive osteoclast surface along the bone surface as well as osteoclast number was measured and expressed relative to the total bone surface measured. Osteoblasts counts were performed by cellular morphology and consisted of cuboidal cells in direct contact with the bone surface. The ratio of osteoclast number to osteoblast number (OC/OB ratio) was determined. The number of adipocytes in the bone marrow were measured using ImageJ (National Institute of Health, Bethesda, MD). Quantitation was performed blinded.

### Statistical analysis

All measurements are presented as the mean ± SEM. Power analyses indicate that an n of 14 is sufficient to detect a 5% difference between groups (with an α of 0.05 and a power of 80%) in bone measures. Significant outliers were removed using the Grubb's test for outliers. One-way ANOVA, with a Fischer *post-hoc* test as well as Pearson Correlations were performed using GraphPad Prism software version 6 (GraphPad, San Diego, CA, USA). *p*-values of < 0.05 were considered significant and values of < 0.01 were considered highly significant.

## Results

Broiler chickens at 7 days of age were infected enterically with *Salmonella* and supplemented with MDY (in the bird feed) beginning at day 1 (continuous) or day 14 (acute) (Figure 1A) to determine if MDY could improve chicken bone health. We infected the birds at this early stage of rapid growth since it is a time when the birds can be more susceptible to infection (Kebede, 2010) and under rapid growth the birds may exhibit a greater bone response to modeling dysregulation. At the time of necropsy, treatments did not appear to adversely affect body condition as there were no significant differences in body mass (Figure 1B) or femur length (data not shown) among groups. As expected, *Salmonella* levels were elevated in the ceca of the infected chickens (Figure 1C). It should be noted that while *Salmonella* was not detected in 22 out of the 24 control birds, 2 out of 24 control birds were positive for the presence of *Salmonella*, but the levels were barely detectable and well below levels seen in infected birds.

**Figure 3:**
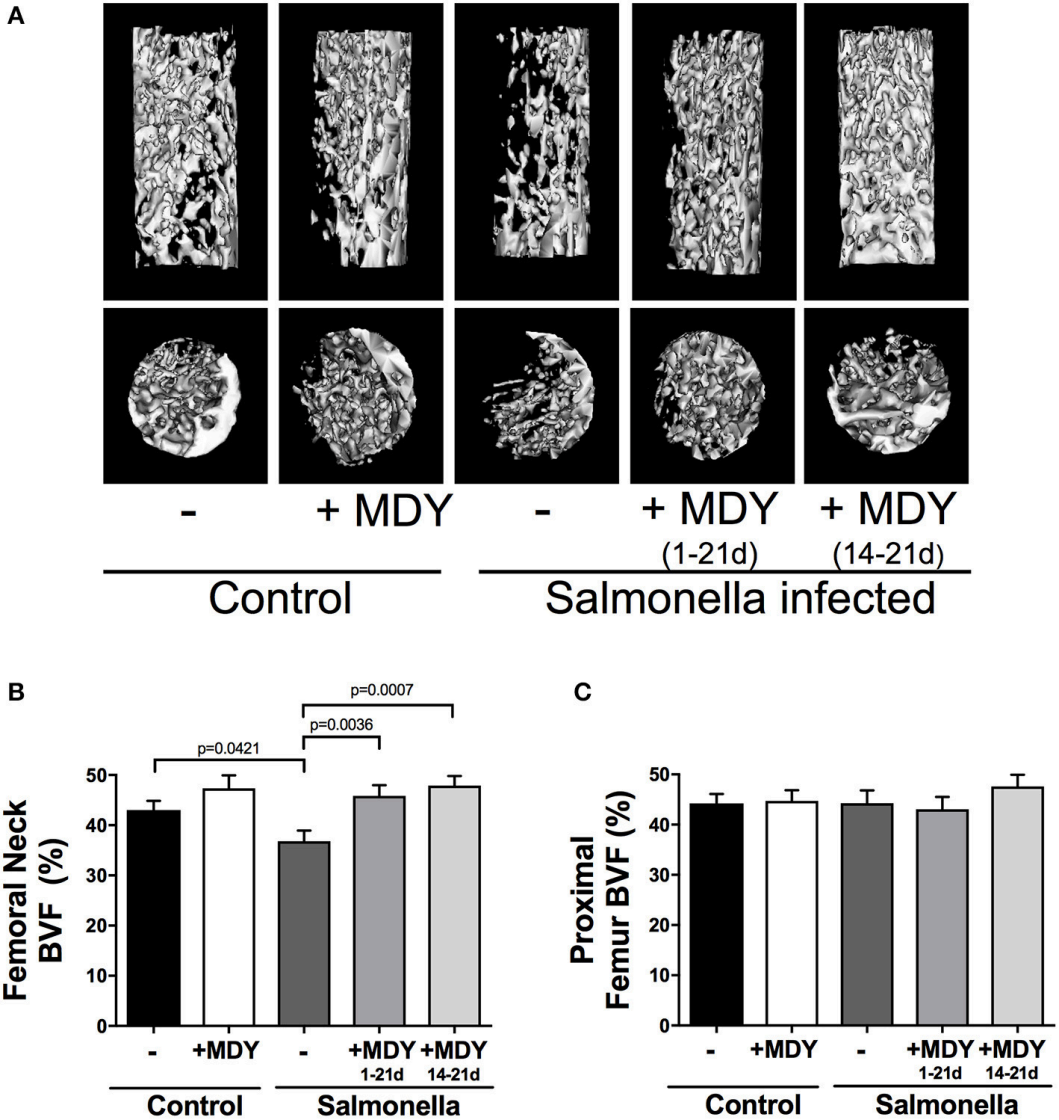
*Salmonella*-induced bone loss specifically in the femoral neck region is prevented by MDY treatment. **(A)** Representative longitudinal and vertical isosurfaces of the chick femoral neck region. **(B)** Bone volume fraction (BVF) of the femoral neck. **(C)** Bone volume fraction of the proximal femur metaphysis. Values are means ± SE. *N* ≥ 15 per group. Lines represent statistical differences (*p* < 0.05) between bars at ends of line as determined by 1-way ANOVA with a Fischer *ad hoc* test.

To examine bone density, we first focused on trabecular bone which is the most metabolically active bone region, and therefore is highly responsive and likely to show changes. Focusing on the femoral neck (Figure 2), where fractures often occur in chickens, we observed clear differences in trabecular bone structure between conditions as seen in the representative isosurface images (longitudinal and cross-sectional) shown in Figure 3A. Quantitation of the femoral neck trabecular bone volume fraction (BVF) revealed a nearly 20% decrease in *Salmonella* infected birds. More importantly, infected birds that were treated with MDY, either from days 1–21 or from days 14–21, were protected from the *Salmonella*-induced bone loss (Figure 3B). Interestingly, femoral neck BVF in control birds treated with MDY trended to be increased relative to untreated controls, suggesting that MDY may support bone health in control animals. To test if the response was region specific, we examined the femoral metaphysis region and found that *Salmonella* infection did not reduce bone volume (Figure 3C). This finding implicates the femoral neck region as being more metabolically active and responsive to *Salmonella* infection and MDY treatment.

**Figure 4:**
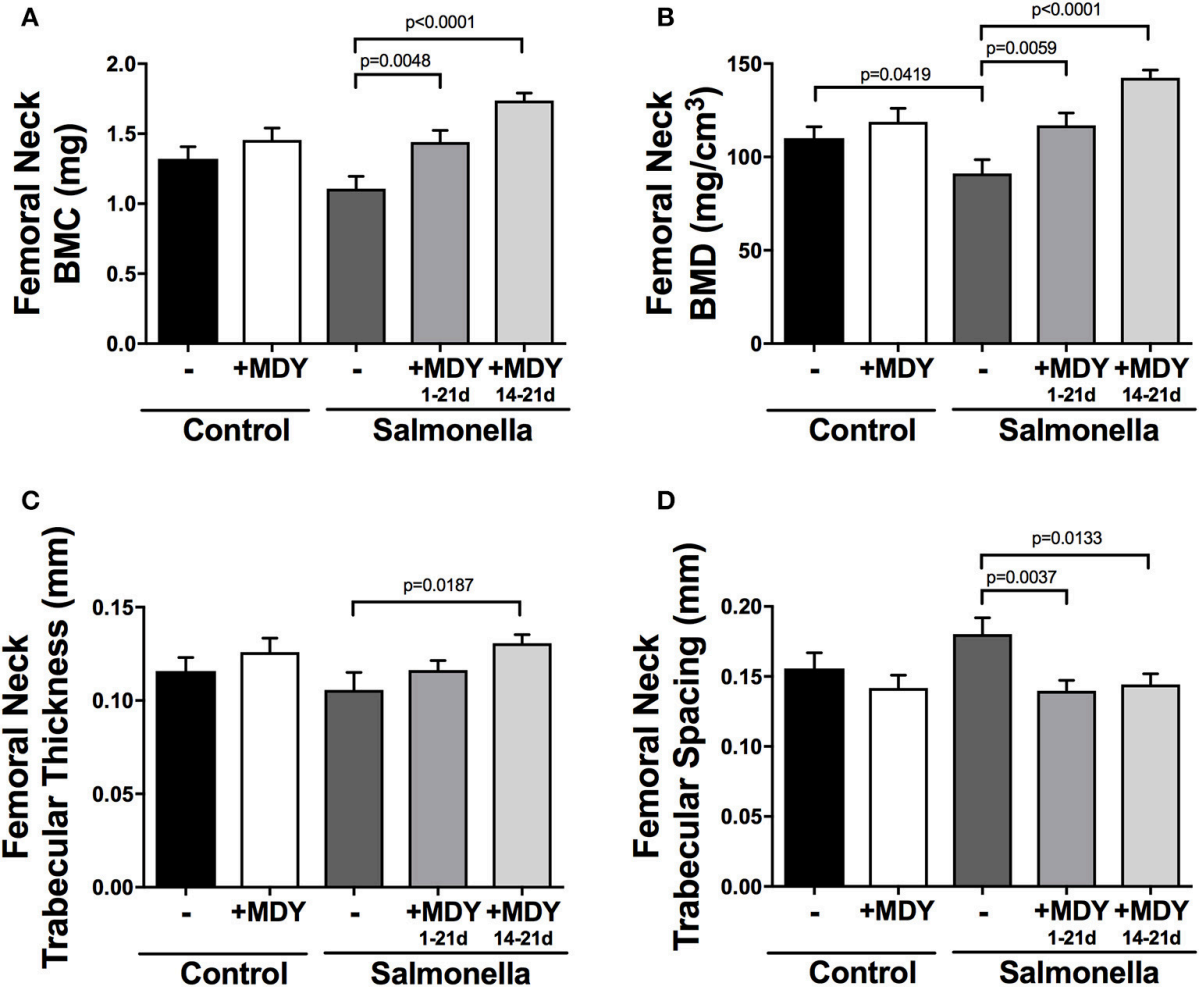
Femoral neck trabecular bone parameters support MDY benefit to bone health in *Salmonella*-infected chicks. Shown are femoral neck trabecular bone parameters: **(A)** Bone mineral content (BMC), **(B)** Bone mineral density (BMD), **(C)** Trabecular thickness and **(D)** Trabecular spacing. Values are means ± SE. *N* ≥ 15 per group. Lines represent statistical differences (*p* < 0.05) between bars at ends of line as determined by 1-way ANOVA with a Fischer *ad hoc* test.

Additional measures of the femoral neck trabecular bone support the bone volume data. Specifically, bone mineral density (BMD) is decreased in *Salmonella-*infected birds and BMD and bone mineral content (BMC) are significantly increased in MDY-treated *Salmonella*-infected birds (Figures 4A,B). The trabecular bone architecture is also altered by infection resulting in trends to decrease trabecular thickness and correspondingly increase spacing between bone trabeculae, while treatment with MDY prevents the negative changes (Figures 4C,D). No changes were observed in trabecular number, indicating that a reduction of trabecular thickness is the predominant response to *Salmonella* infection in birds.

**Figure 5:**
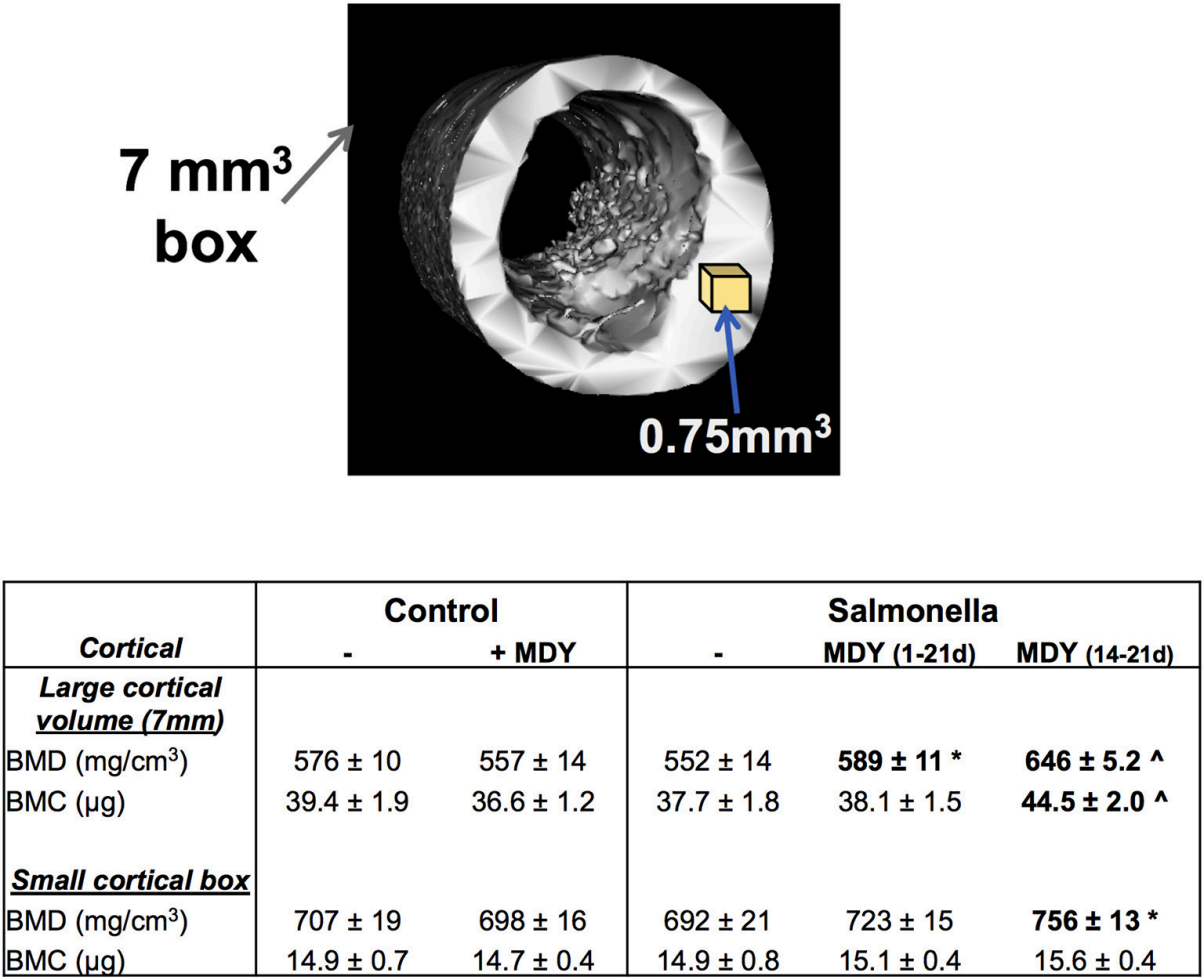
MDY treatment benefits cortical area and density. Top image is a representative isosurface of chick diaphyseal cortical bone demonstrating the 7 mm cube volume region and indicating where the small cortical cube (0.7 mm^3^) region is taken. Table represents measures as means ± SE. *N* ≥ 15 per group. ^*^p < 0.05 compared to *Salmonella* control; ^∧^*p* < 0.01 compared to *Salmonella* control as determined by 1-way ANOVA with a Fischer *ad hoc* test.

Next we examined the cortical bone of the femoral diaphysis. Though cortical BMD and BMC appear lower in *Salmonella*-infected birds than controls, the levels were not significantly different (Figure 5). This was true for other cortical bone parameters such as inner and outer perimeters, cortical area, marrow area, etc. (not shown), consistent with this region having less metabolic/modeling activity. However, MDY treatment, both the 1–21 and 14–21 day treatments, increased cortical BMD in *Salmonella*-infected birds (Figure 5).

**Figure 6:**
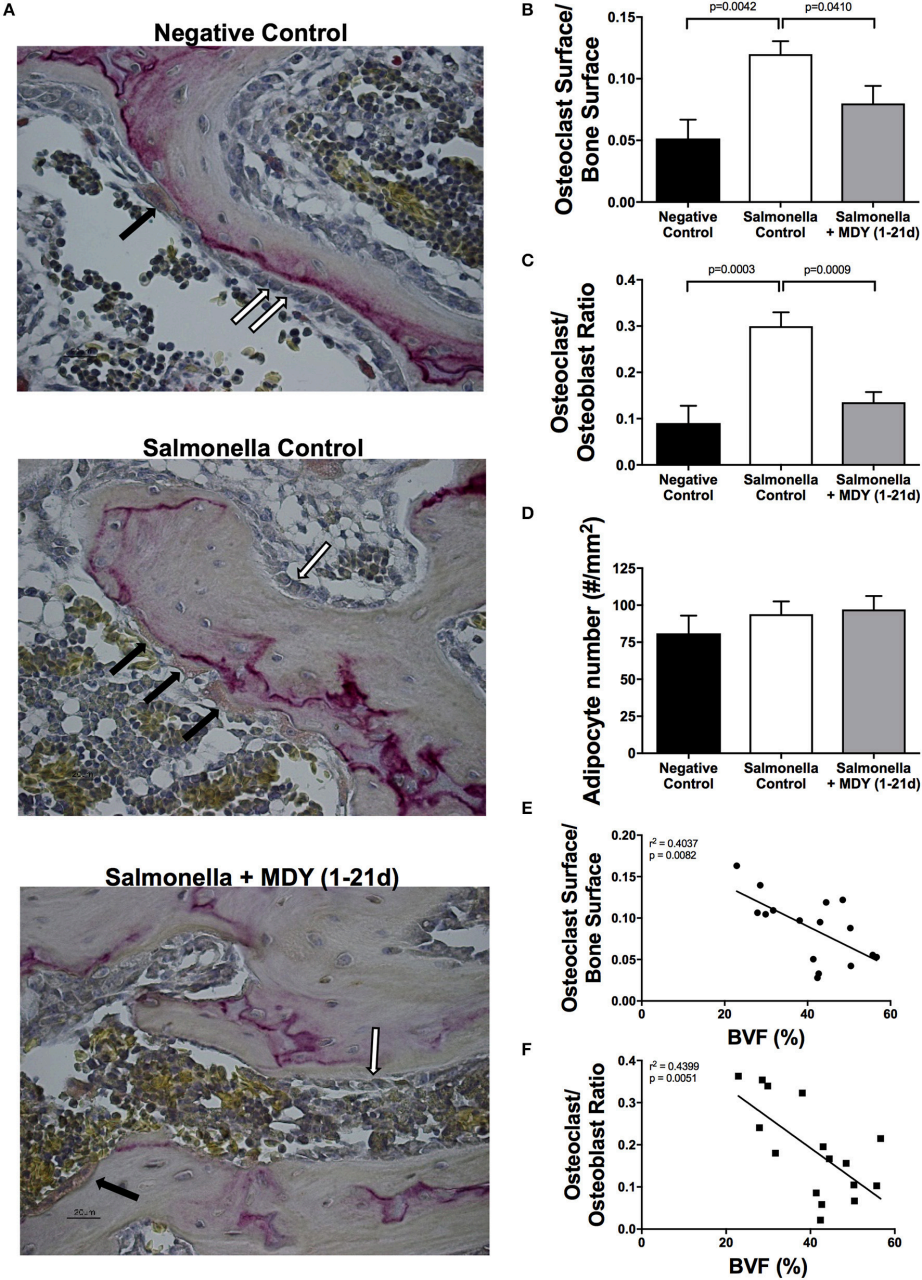
MDY prevents *Salmonella*-induced increase in osteoclast surface and osteoclast to osteoblast ratio. **(A)** Representative TRAP-stained, 40X images of the femoral neck region. Black arrows indicate TRAP-positive osteoclasts; White arrows indicate cuboidal osteoblasts. **(B)** Ratio of osteoclast surface over total bone surface measured in chick femurs stained with TRAP. **(C)** Ratio of osteoclast to osteoblast number. **(D)** Quantification of bone marrow adipocytes. **(E,F)** Pearson Correlation analyses of osteoclast surface/bone surface or osteoclast/osteoblast number to bone volume fraction (BVF). Values are means ± SE. *N* = 6 per group. Lines represent statistical differences (*p* < 0.05) between bars at ends of line as determined by 1-way ANOVA with a Fischer *ad hoc* test.

As decreased bone volume can be a consequence of either increased resorption by osteoclasts or decreased formation by osteoblasts, we sought to examine the effect of *Salmonella* infection on these cell populations in the femoral neck using histomorphometry measures. Consistent with increased resorption, *Salmonella*-infected birds displayed an increase in TRAP-positive osteoclast surface with respect to both control and MDY-treated *Salmonella*-infected birds (Figures 6A,B). Osteoblast surface did not significantly change (data not shown). However, there was a significant increase in the ratio of osteoclast to osteoblast surface along the femoral neck trabecular bone in the *Salmonella*-infected birds relative to both control and MDY-treated *Salmonella*-infected birds (Figure 6C). Both the osteoclast surface as well as the ratio of osteoclasts to osteoblasts were significantly correlated to bone volume fraction (BVF%) (Figures 6E,F). Quantiation of marrow adipocytes (derived from mesenchymal stem cells and thought to be inversely proportional to osteoblast numbers) revealed no significant difference between groups (Figure 6D), consistent with no observed change in osteoblast number.

## Discussion

While most poultry in the US have been grown under chronic sub-therapeutic antibiotic treatment, there has been a push for antibiotic-free chickens to reduce the risk of developing antibiotic resistant bacteria and to promote chemical-free chicken meat. With stressfull housing and the removal of chronic antibiotic treatment, chickens are more susceptible to infection (Cook, 2000). The inflammatory state may be an important contributor to decreased skeletal health (McNamee et al., 1999; Corr et al., 2003; Huff et al., 2006; Tellez et al., 2014; Wideman, 2016; Bielke et al., 2017) as suggested by mouse studies (Mccabe et al., 2013; McCabe et al., 2015; Irwin et al., 2016; Collins et al., 2017a,b; Rios-Arce et al., 2017; Schepper et al., 2017). The bone deformation or loss can result in fractures that cause the chickens to be culled, which costs the industry hundreds of millions of dollars annually (Cook, 2000). Here we show that *Salmonella* infection, on its own, is sufficient to cause broiler chicken femoral neck bone loss. We further demonstrate that MDY is an effective treatment to prevent broiler chicken bone loss.

Researchers indicate that up to 30% of broiler chickens suffer from gait defects (Knowles et al., 2008; Dinev, 2009). The femur is a key sight of bone degeneration that contributes to difficulty walking (McNamee and Smyth, 2000; Dinev, 2009; Durairaj et al., 2009; Olkowski et al., 2011). Olkowski et al. (2011) demonstrated that 85–90% of broiler chickens showing signs of lameness have pathology in the proximal end of the femur that typically involves changes in the femoral head including loss of subchondral bone, less organic matrix content, and in severe cases fractures. The results of these and similar studies led us to study *Salmonella* effects specifically on the femur. Our studies indicate that the femoral neck displays significant bone loss, changes in trabecular bone architecture, and an increase in the bone surface covered by osteoclasts as well as the ratio of osteoclasts to osteoblasts in *Salmonella-*infected birds. Interestingly, we saw no difference in bone density at the proximal femoral metaphysis or the femoral diaphysis. This location specificity is important to note, since benefits of other therapies on broiler chicken skeletal health may be missed if only proximal trabecular bone is examined. While we were not able to analyze other bones such as vertebrae, to determine if bone loss occurs at other skeletal sites, our findings are important because they demonstrate that infection and treatments can have varying responses within sites of a single bone.

Rodent disease models display bone loss in response to bacterial intestinal infection as well as inflammation secondary to colitis (Lin et al., 1996; Harris et al., 2009; Irwin et al., 2013). Our lab has also shown that low levels of inflammation from a surgical procedure (dorsal skin incision) is enough stimulus to cause bone loss in mice (Collins et al., 2016). Physiologic inflammation is typically controlled by the body to maintain homeostasis. However, activation of an immune response as seen in infection, can lead to the production of proinflammatory cytokines and alterations in the immune cell population that are important for reducing further infection but simultaneously can reduce anabolic processes such as bone formation (Harris et al., 2009). Interestingly, unlike previous reports which indicate that decreased bone formation is the main cause for bone loss in models of intestinal inflammation (Lin et al., 1996; Harris et al., 2009), *Salmonella* infection in this study lead to an increase in the number of multi-nucleated TRAP-positive osteoclasts on the bone surface. This finding points toward an increase in bone resorption as the cause for decreased bone density in *Salmonella*-infected chickens. Accordingly, inflammation, as would be seen with infection or colitis, is known to promote osteoclastogenesis.

Past studies have indicated a beneficial role for oral probiotics in reducing broiler chick lameness (Wideman and Prisby, 2012), though exact mechanisms are not known. Here we show that oral ingestion of MDY also benefits broiler bone health. In this study we chose two different treatment schedules, continuous (1–21 days) and acute (14–21 days), in order to determine if MDY was able to prevent bone loss or if it could reverse bone loss. We found that continous MDY treatment counteracts femoral neck bone loss, structural changes and the increase in osteoclast surface caused by *Salmonella* infection. Even more impressive is our finding that acute MDY treatment, beginning 7 days after *Salmonella* infection, was able to prevent the decreased bone density in both the femoral neck as well as cortical bone. We did not measure bone density 1 week after *Salmonella* infection. If presumably adverse bone changes were occurring within the first week of infection, MDY treatment may be able to reverse bone loss. Future studies will investigate the latter postulate on MDY efficacy. Additionally, it will be important for future studies to examine longer-term effects of infection on the chicken skeleton. For example, if we extended the time course of the current study, infected chickens may continue to display increased bone loss (which could affect the entire femur bone trabecular structure); however, it is also possible that with time the bone loss we observe is regained following control of the infection.

While we do not know the exact mechanisms accounting for the beneficial effects of MDY on chicken bone health, our data indicate that MDY does not work by eradicating *Salmonella*. This is demonstrated by the high Salmonella recovery rates from the ceca of MDY treated birds. This suggests, that MDY may be preventing the negative gut-bone signaling in responses to Salmonella infection. While our findings indicate that altered regulation of osteoclast activity contributes to the mechanism of *Salmonella*-induced bone loss and to MDY prevention of bone loss, additional analyses are needed (such as intestinal cytokine profiles, histology and intestinal permeability) to identify the specific mechanisms by which MDY influences the gut to cause the changes in bone. Gut inflammation, altered microbiota composition and increased barrier function are several key intestinal parameters that contribute to bone loss in mammals (Lin et al., 1996; Sylvester, 2005; Hamdani et al., 2008; Harris et al., 2009; Irwin et al., 2013, 2016; Mccabe et al., 2013; McCabe et al., 2015; Sylvester et al., 2013; Collins et al., 2017a,b; Rios-Arce et al., 2017; Schepper et al., 2017) and in poultry (Huff et al., 2006; Tellez et al., 2014; Wideman, 2016; Bielke et al., 2017). Past studies indicate that PEG derivatives can benefit intestinal health by increasing barrier function, reduce intestinal inflammation and contributing to overall host health (Wu et al., 2004; Valuckaite et al., 2013). Therefore, based on the above reports, it is likely that MDY influences the enteric health through multiple mechanisms that involve enhancing barrier function and reducing inflammation. Taken together, our work and the work of others indicate that orally ingested MDY prevents and possibly reverses the *Salmonella*-induced, negative effects on femoral bone density and architecture in broiler chickens and consequently be an effective way to promote chick bone health.

## Author contributions

SR, BH, VK, LB, and LM have nothing to disclose. RP holds financial interest in Midway Pharmaceuticals, Inc. and Midway Animal Sciences. Midway Pharmaceuticals, Inc. and Midway Animal Sciences contributed MDY and methods of dosing and delivery but were not involved in the overall study design or collection, analysis or interpretation of the data.

### Conflict of interest statement

RP holds financial interest in Midway Pharmaceuticals, Inc. and Midway Animal Sciences. The other authors declare that the research was conducted in the absence of any commercial or financial relationships that could be construed as a potential conflict of interest.
